# Phosphatidylinositol binding of *Saccharomyces cerevisiae* Pdr16p represents an essential feature of this lipid transfer protein to provide protection against azole antifungals

**DOI:** 10.1016/j.bbalip.2014.07.014

**Published:** 2014-10

**Authors:** Roman Holič, Zuzana Šimová, Tim Ashlin, Vladimír Pevala, Katarína Poloncová, Dana Tahotná, Eva Kutejová, Shamshad Cockcroft, Peter Griač

**Affiliations:** a Department of Membrane Biochemistry, Institute of Animal Biochemistry and Genetics, Slovak Academy of Sciences, 900 28 Ivanka pri Dunaji, Slovakia; b Department of Neuroscience, Physiology and Pharmacology, Division of Biosciences, University College London, London WC1E 6JJ, United Kingdom; c Department of Biochemistry and Structural Biology, Institute of Molecular Biology, Slovak Academy of Sciences, 845 51 Bratislava, Slovakia

**Keywords:** PI, Phosphatidylinositol, PC, Phosphatidylcholine, LPA, Lysophosphatidic acid, LPC, Lysophosphocholine, PI4P, Phosphatidylinositol (4) phosphate, PI3P, Phosphatidylinositol (3) phosphate, PI5P, Phosphatidylinositol (5) phosphate, PE, Phosphatidylethanolamine, S1P, Sphingosine 1-Phosphate, PI3,4P_2_, Phosphatidylinositol (3,4) bisphosphate, PI3,5P_2_, Phosphatidylinositol (3,5) bisphosphate, PI4,5P_2_, Phosphatidylinositol (4,5) bisphosphate, PI3,4,5P_3_, Phosphatidylinositol (3,4,5) trisphosphate, PA, Phosphatidic acid, PS, Phosphatidylserine, NPPMs, nitrophenyl(4-(2-methoxyphenyl)piperazin-1yl)methanone, PITP, phosphatidylinositol transfer protein, HL60, human promyelocytic leukemia cells, Osh, oxysterol-binding homology, MIC, minimal inhibitory concentration, Lipid binding, *Saccharomyces cerevisiae*, Azole resistance, *SFH3*, Sterol metabolism

## Abstract

Pdr16p is considered a factor of clinical azole resistance in fungal pathogens. The most distinct phenotype of yeast cells lacking Pdr16p is their increased susceptibility to azole and morpholine antifungals. Pdr16p (also known as Sfh3p) of *Saccharomyces cerevisiae* belongs to the Sec14 family of phosphatidylinositol transfer proteins. It facilitates transfer of phosphatidylinositol (PI) between membrane compartments in *in vitro* systems. We generated Pdr16p^E235A, K267A^ mutant defective in PI binding. This PI binding deficient mutant is not able to fulfill the role of Pdr16p in protection against azole and morpholine antifungals, providing evidence that PI binding is critical for Pdr16 function in modulation of sterol metabolism in response to these two types of antifungal drugs. A novel feature of Pdr16p, and especially of Pdr16p^E235A, K267A^ mutant, to bind sterol molecules, is observed.

## Highlights

• Yeast Pdr16p binds phosphatidylinositol (PI) and cholesterol in lipid binding assay.
• Pdr16p^E235A, K267A^ is defective in PI binding, it binds sterols instead of PI.
• Pdr16p defective in PI binding does not fulfill Pdr16p role in azole protection.
• PI binding of Pdr16p is critical for its function.

## 1 Introduction

Azole antifungals are often the primary choice in treating fungal infections. Yeast and fungi are able to develop resistance to counteract the action of azoles. Four major mechanisms of resistance to azoles have been described: (i) decreased drug concentration mostly by upregulation of drug efflux pumps, (ii) alterations of the target enzyme, lanosterol C14α-demethylase, (iii) upregulation of the target enzyme, and (iv) changes in sterol biosynthesis compensating for the block in the ergosterol biosynthetic pathway caused by azoles (reviewed in [1], [2], [3]). Pdr16p emerged recently as a factor of clinical azole resistance in fungal pathogens of humans. The best documented case is Pdr16p of *Candida albicans* [4], [5]. In many azole resistant clinical isolates of *C. albicans* the *PDR16* gene was overexpressed in addition to multidrug transporters [4]. Deletion of Ca*PDR16* in azole-resistant clinical isolates decreased their resistance to azoles approximately two-fold [4], [5]. Overexpression of Ca*PDR16* resulted in yeast cells approximately two-fold more resistant to fluconazole compared to parental, azole-susceptible cells. These results implicate Pdr16p in low-level resistance of *C. albicans* to azoles [4]. In another clinically important opportunistic yeast pathogen, *Candida glabrata*, *pdr16Δ* mutation also increased the susceptibility of yeast cells to azole antifungals and reduced cell surface hydrophobicity and biofilm production [6]. In addition, *Saccharomyces cerevisiae* Pdr16p is an important part of the mechanism responsible for the development of evolutionary fluconazole resistance based on the observation that *PDR16* deletion strongly reduced the ability of yeast cells to develop this type of azole resistance [7]. Taken together, Pdr16p could be considered as one of the targets in preventing adverse azole resistance in fungi.

Pdr16p (also called Sfh3p) is a member of the yeast *S. cerevisiae* Sec14-like phosphatidylinositol transfer protein (PITP) family [8], [9]. The most pronounced phenotype of the *pdr16Δ* cells is their increased susceptibility to all azoles tested [10], [11], [12]. This hypersensitivity of the *pdr16Δ* cells is not a typical multidrug resistance phenomenon as *pdr16Δ* cells do not display increased susceptibility towards any of the other drugs tested, such as nystatin, cycloheximide, rhodamine-6G, oligomycin, 4-nitroquinoline-N-oxide, antimycin A, ethidium bromide, and crystal violet [12]. In the presence of azole antifungals, *pdr16Δ* strain accumulated increased levels of the yeast sterol biosynthetic pathway precursors, lanosterol and squalene, at the expense of the final product, ergosterol, when compared to its parental wild-type strain [11]. It was also shown that the increased susceptibility of the *pdr16Δ* strain to azoles and the enhanced changes in sterol biosynthesis upon exposure to azoles are not due to the increased intracellular concentrations of azoles in the *pdr16Δ* cells [11]. It remains to be established, however, whether the role of Pdr16p in conferring resistance to azole antifungals is direct or whether it is mediated *via* some signaling role of the Pdr16p.

The founding member of the PITP family in the yeast *S. cerevisiae*, Sec14p, is required for transport of secretory proteins from the Golgi complex and is essential for cell viability [13], [14]. Trafficking and proper localization of lipid raft proteins were suggested as a major function of Sec14p [15]. Sec14p facilitates the transfer of PI and phosphatidylcholine (PC) between donor and acceptor membranes in *in vitro* assays [9], [16]. Pdr16p differs significantly from Sec14p: (i) it is able to transfer PI and not PC in *in vitro* systems [9], (ii) it localizes to lipid particles and the cell periphery, compared to the predominantly cytosolic localization of Sec14p [17], (iii) its overexpression does not rescue *sec14^ts^* mutant lethality at non-permissive temperature [9], [17]. Recently the solved crystal structure of Pdr16p points to another major difference between Sec14p and Pdr16p. Apo-Pdr16p forms a dimer through the hydrophobic interactions of gating helices [18], [19] while two other members of the Sec14-like family of yeast PITPs for which the crystal structure is known, Sec14p [20] and Sfh1p [21], crystallize in monomeric forms. Binding of PI by Pdr16p leads to dissociation of the dimer into monomers which are considered to be the physiologically-active form of Pdr16p [18], [19].

To address the question to which extent the PI binding ability of Pdr16p is relevant to its function in relation to azole resistance, we generated Pdr16p^E235A, K267A^ mutant defective in PI binding. We show that this PI binding-deficient mutant is not able to fulfill the role of Pdr16p in providing protection against azole antifungals, establishing thus PI binding as an essential feature of Pdr16p.

## 2 Materials and methods

### 2.1 Media and chemicals

Media components were obtained from Becton-Dickinson (USA) or BioLife (Italy). Miconazole was from MP Biomedicals (USA), terbinafine and amorolfine were from Sigma-Aldrich (USA). [1-^14^C] acetic acid was purchased from American Radiolabeled Chemicals (UK). Fine chemicals were mostly from Sigma-Aldrich (USA) or MP Biomedicals (USA).

### 2.2 Strains and culture conditions

Wild-type *S. cerevisiae* strain FY1679-28c and from its derived *pdr16Δ* strain originally from A. Goffeau laboratory (Catholic University Louvain, Belgium) [12] were kindly provided by G. Daum (Technical University, Graz, Austria). Episomal plasmid containing *PDR16* under its own promoter was constructed on the basis of a 2 μm plasmid YEplac181 [22]. Details of its construction are described in [17]. To create a *PDR16* allele encoding a protein defective in PI binding, plasmid YEplac181-*PDR16* was modified using *in vitro* site-directed mutagenesis protocol based on Agilent Site-Directed Mutagenesis Kit (Agilent, USA) with minor modifications. Briefly, PCR primers containing desired mutations (E235A, 5′-CTCATTATCCAGCAAGACTAGGAAAAGCAC-3′ and K267A, 5′-GTTCATCAAAAACTAGCGCTTCACGGGTC-3′, changes underlined) were used to generate a DNA fragment (126 bp) using plasmid YEplac181-*PDR16* as template. This DNA fragment served as a primer in the whole plasmid PCR extension creating mutated plasmid YEplac181-*PDR16*^*E235A*,^ *^K267A^*. Parental non-mutated methylated DNA was cleaved with *DpnI* restriction enzyme. The PCR product was subsequently transformed into *E. coli*, mutated plasmid was isolated and correct insertion of desired nucleotide changes confirmed by DNA sequencing. To create plasmids for recombinant protein production in *E. coli* ORFs *PDR16* and *PDR16*^*E235A*,^ *^K267A^* were amplified from plasmids YEplac181-*PDR16* and YEplac181-*PDR16*^*E235A*,^ *^K267A^* using the following primers: 5′-TGTACCATATGTTCAAGAGATTTAGCAAAAAG-3′ and 5′-GACGTCTCGAGGCGGCCGCCACGGTACTGCTTTCCGA-3′, *Nde*I and *Not*I sites are underlined. Amplified ORFs were cut with *Nde*I and *Not*I restriction enzymes and inserted into the corresponding sites of pET26 vector (Merck, USA) to create plasmids pET26-*PDR16*-6xHis and pET26-*PDR16*^*E235A*,^ *^K267A^*-6xHis. All constructs containing PCR amplified DNA fragments were checked by DNA sequencing. *Escherichia coli* Rosetta strain (F^-^*omp^T^ hsdS_B_ (*r_B_^-^m_B_^-^) *gal dcm* (DE3) pRARE (*Cam^R^*) was from Merck.

Yeast strains were grown on yeast extract/peptone/dextrose (YEPD; 2% glucose) media unless otherwise stated. Yeast strains containing episomal plasmids were maintained and pre-grown on standard synthetic minimal medium (0.67% YNB without amino acids, 2% glucose) supplemented with essential amino acids and bases as required for plasmid maintenance.

### 2.3 Drugs susceptibility testing

Drug susceptibility was determined by a spot assay. Yeast cultures were pre-grown overnight in YNB-LEU media, diluted and spotted as 10-fold dilutions onto YEPD solid media containing miconazole, terbinafine, or amorolfine. The following concentrations of drugs were used: miconazole 2, 5, 10, 20, 40, 60, 80 ng/ml, terbinafine 0.5, 1, 2, 5, 10, 30 μg/ml, amorolfine 0.5, 1, 2, 5, 8, 10 ng/ml. Drugs were added to the growth media before plate pouring from 1000 × stock solution in DMSO. The growth was scored after 2 days of incubation at 28 °C. Minimal inhibitory concentration (MIC) was determined as the lowest concentration of a drug that inhibited the visible growth of the last two dilutions on YEPD plates after 2 days of incubation at 28 °C.

### 2.4 Protein expression and purification

His_6_ C-terminally tagged Pdr16p proteins were purified from *E. coli* (Rosetta strain, Merck) transformed with plasmids pET26-*PDR16*-6xHis and pET26-*PDR16*^*E235A*,^ *^K267A^*-6xHis, respectively. Expression of recombinant proteins was induced with 1 mM IPTG. Bacterial lysates in 50 mM phosphate buffer pH 8.0 containing 300 mM NaCl were loaded onto Ni-NTA agarose columns (Qiagen) and washed repeatedly with 50 mM imidazole in 50 mM phosphate buffer, 300 mM NaCl, pH 8.0. His-tagged proteins were eluted with 200 mM imidazole in 50 mM phosphate buffer, 300 mM NaCl, pH 8.0 [23].

### 2.5 Size exclusion chromatography

Purified recombinant Pdr16 proteins were subjected to size exclusion chromatography on a Superose™12 10/300 GL column (GE Healthcare) equilibrated with 20 mM PIPES pH 6.8, 250 mM NaCl, 2.7 mM KCl buffer. Proteins were monitored at 280 nm. The flow rate was 0.4 ml/min.

### 2.6 Lipid–protein overlay assay

Echelon PIP 6001 strips with phospholipids immobilized on nitrocellulose membranes were first incubated for 1 h in 3% (wt/vol) fatty acid-free BSA (Sigma-Aldrich) in TBST (50 mM Tris–HCl, pH 7.5, 150 mM NaCl, and 0.1% (vol/vol) Tween 20) to block unspecific interactions. They were then incubated overnight at 4 °C with 4 ml of TBS buffer containing 0.1 μg/ml of recombinant purified Pdr16-His and Pdr16^E235A, K267A^-His proteins, respectively. Membranes were then incubated for 2 h with 1:1000 dilution of anti-His antibody (Qiagen) followed by 2 h incubation with 1:20,000 dilution of secondary alkaline phosphatase conjugated anti-mouse IgG (Sigma-Aldrich). Repeated washing steps with TBS containing 0.05% Tween 20 were inserted between all incubation steps.

### 2.7 Binding of cellular lipids

Association of eukaryotic cellular lipids with the PITPs was analyzed as described previously [23], [24]. In brief, HL60 cells were labeled with 1 μCi/ml [^14^C] acetate in RPMI 1640 medium for 48 h. The cells were permeabilized with streptolysin O, and the leaked cytosol was removed by centrifugation. Permeabilized cells (~ 10^7^ cells) were incubated with 120 μg of the respective recombinant protein (100 μl) for 20 min at 37 °C in the presence of 2 mM Mg^2 +^-ATP and 100 nM Ca^2 +^ buffered with 3 mM EGTA. A sample of the protein was saved and run on 12% SDS-PAGE. At the end of the incubation, the cells were removed by centrifugation, and the recombinant proteins in the supernatant were captured on nickel beads. An aliquot of the recovered proteins was run on SDS-PAGE to assess their recovery, and the rest of the sample was used for lipid extraction. The lipids were resolved by thin layer chromatography using a Whatman silica gel 60 TLC plate using chloroform/methanol/acetic acid/water (75:45:3:1, vol/vol) as the mobile phase. Lipids extracted from the permeabilized HL60 cells (approximately 100,000 dpm) were analyzed alongside for comparison. The TLC plates were exposed to Fuji phosphorimaging screens and analyzed using a Fuji BAS1000 phosphorimaging system. Both the SDS-PAGE and TLC images were analyzed using AIDA software. Control recombinant Sec14p was kindly provided by V. Bankaitis (Texas A&M Health Science Center, Texas, USA), and recombinant PITPα was prepared as described previously [25].

### 2.8 Lipid extraction and analysis

Non-saponifiable lipids for sterol analysis by HPLC were isolated by the modified procedure of Breivik and Owades [26]. Briefly, 1 × 10^9^ cells, broken by homogenization with glass beads, were incubated in 3 ml of 60% KOH (wt/vol) in 50% methanol (vol/vol) for 2 h at 70 °C. Non-saponifiable lipids were extracted twice with 3 ml of *n*-hexane and combined extracts were dried under a stream of nitrogen. The lipid residue was dissolved in acetone and analyzed by reverse phase HPLC on Agilent 1100 instrument equipped with Eclipse XDB-C8 column (Agilent Technologies, USA), diode array detector (Agilent Technologies, USA) and Corona charged aerosol detector (ESA Inc., USA). Sterols were eluted at 40 °C with 95% methanol at flow rate 1 ml/min. Peak identity was determined from the retention times of standards — ergosterol, lanosterol (Serva, Germany) and squalene (Sigma-Aldrich, USA) and from their characteristic spectra. Sterol quantity was calculated from calibration curves constructed for individual standards.

## 3 Results

### 3.1 Generation of the Pdr16p^E235A, K267A^ mutant

**Fig. 1:**
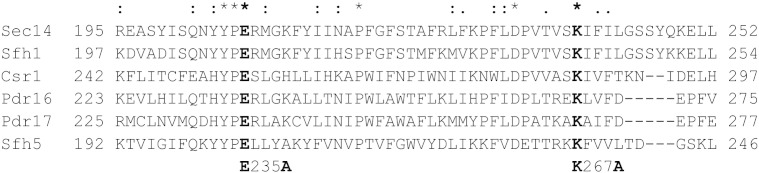
Alignment of the Sec14 group of yeast proteins. Highly homologous C-terminal regions of Sec14p and its 5 yeast homologues were aligned using the ClustalW2 program (http://www.ebi.ac.uk/). Asterisks (**^⁎^**) indicate conserved residues, colons (:) and periods (.) indicate strongly and loosely homologous residues, respectively. Numbers indicate amino acid residues of respective proteins. Conserved amino acids E235 and K267 (in bold) were changed to alanine to create Pdr16 mutant defective in PI binding. Note that in databases another *SFH1* (ORF YLR321c) is referred. In this study the name *SFH1* is used for ORF YKL091c.

Pdr16p was shown to stimulate transfer of PI between membrane compartments in an *in vitro* system [9]. To investigate the importance of PI binding of Pdr16p in its function to provide protection against azole antifungals we mutated two amino acids, glutamic acid 235 and lysine 267 of Pdr16p to alanine. E235 and K267 of Pdr16p are conserved residues in all yeast Sec14 homologues (Fig. 1). They correspond to amino acids E207 and K239 of Sec14p that were shown previously to be essential for *in vitro* PI transfer activity of Sec14p [27]. A similar approach was used to explore the importance of PI binding/transfer for function of Sfh5p in delivering the exocytic signal [28]. Recently published structures of Pdr16p (Sfh3p) indicate that both these two amino acids, E235 and K267, line the lipid binding cavity of Pdr16p and their side chains protrude into the cavity. Importantly, amino acid K267 in all published structures interacts with the molecule of PI that resides inside the lipid binding cavity [18], [19], [29].

### 3.2 Pdr16p^E235A, K267A^ mutant is defective in PI binding and displays increased ability to bind sterol molecules

**Fig. 2:**
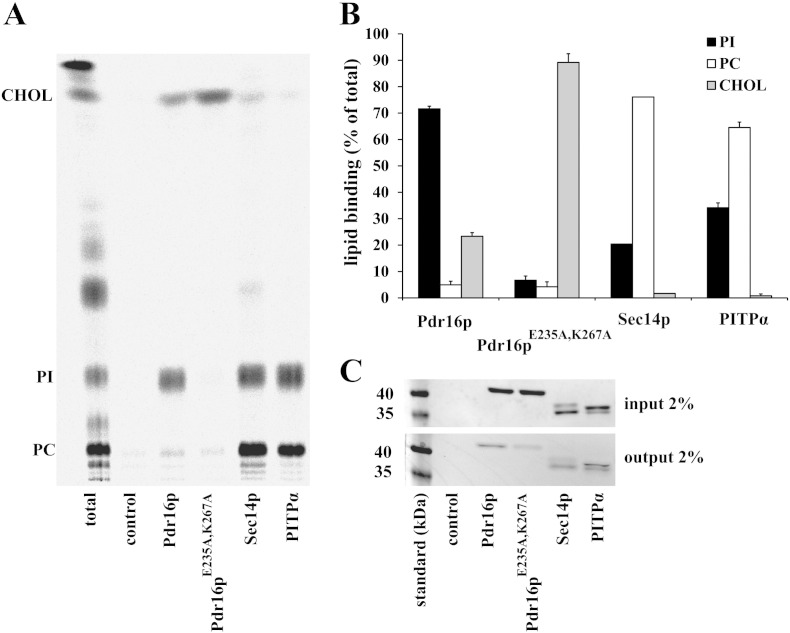
Analysis of the lipid binding specificity of Pdr16p and Pdr16p ^E235A, K267A^. A. Wild-type Pdr16p, Pdr16p ^E235A, K267A^, Sec14p, and PITPα (all at 120 μg — input) were incubated with permeabilized HL60 cells prelabeled with ^14^C-acetate for 48 h. HL60 cells were then removed by centrifugation and His-tagged proteins in the supernatant were re-isolated using nickel beads (output). The lipids bound to the protein were extracted and separated by TLC. “Total” represents portion of lipids extracted from HL60 cells prelabeled with ^14^C-acetate. In “control” no protein was added to HL60 cells. B. Quantification of the lipid bound to lipid transfer proteins expressed as a percentage of total lipid binding (total recovered radioactivity in PC + PI + CHOL) to each protein. Data represent mean ± S.E.M. value from three independent experiments for Pdr16 and Pdr16p ^E235A, K267A^ and two independent experiments for PITPα. Sec14p was assayed once. Abbreviations: PC, Phosphatidylcholine; PI, Phosphatidylinositol; CHOL, Cholesterol. C. To monitor capture of the respective protein by the nickel beads, a sample of the protein (2% of total) was analyzed by SDS-PAGE and stained with Coomassie Blue. A representative figure is shown. Note, that in every independent experiment relatively less Pdr16p ^E235A, K267A^ was recovered compared to Pdr16p.

To test whether the mutated version of Pdr16p, Pdr16p^E235A, K267A^, is able to bind PI we used lipid binding assay using permeabilized HL60 cells [23], [24]. The advantage of this method is that the lipid binding protein can choose from the selection of lipids that are available in permeabilized eukaryotic cells. Radiolabeled permeabilized HL60 cells were co-incubated with recombinant wild-type Pdr16p, with Pdr16p^E235A, K267A^ mutant or with control recombinant Sec14p and/or PITPα. Following co-incubation the respective proteins were re-purified on Ni-NTA affinity columns using C-terminal His-tag, the bound lipids were extracted and analyzed using TLC (see Materials and methods section for details). This lipid binding assay shows that wild-type recombinant Pdr16p binds preferentially PI and cholesterol (Fig. 2). Some radiolabeled PC was also extracted from re-purified Pdr16p. However, it represents only 2–3% of recovered lipid associated radioactivity in case of Pdr16p compared to close to 80% in case of Sec14p control. It remains to be determined whether this small amount of PC associated with Pdr16p represents lipid inside the lipid binding cavity of Pdr16p or whether it represents lipid unspecifically associated with the protein. Mutant Pdr16p^E235A, K267A^ binds almost exclusively cholesterol (95–96% of recovered lipid associated radioactivity) with very little PI and PC present. The surprising ability of Pdr16p and especially of its mutant defective in PI binding, Pdr16p^E235A, K267A^ to bind cholesterol in the *in vitro* lipid binding assay substantiate further study as it represents the first example of a phospholipid binding protein to effectively bind a sterol molecule. Nevertheless, the *in vitro* lipid binding assay demonstrated that mutant Pdr16p^E235A, K267A^ is unable to bind PI effectively and can be used to test whether PI binding is an essential feature of this protein to provide protection against azole antifungals.

**Fig. 3:**
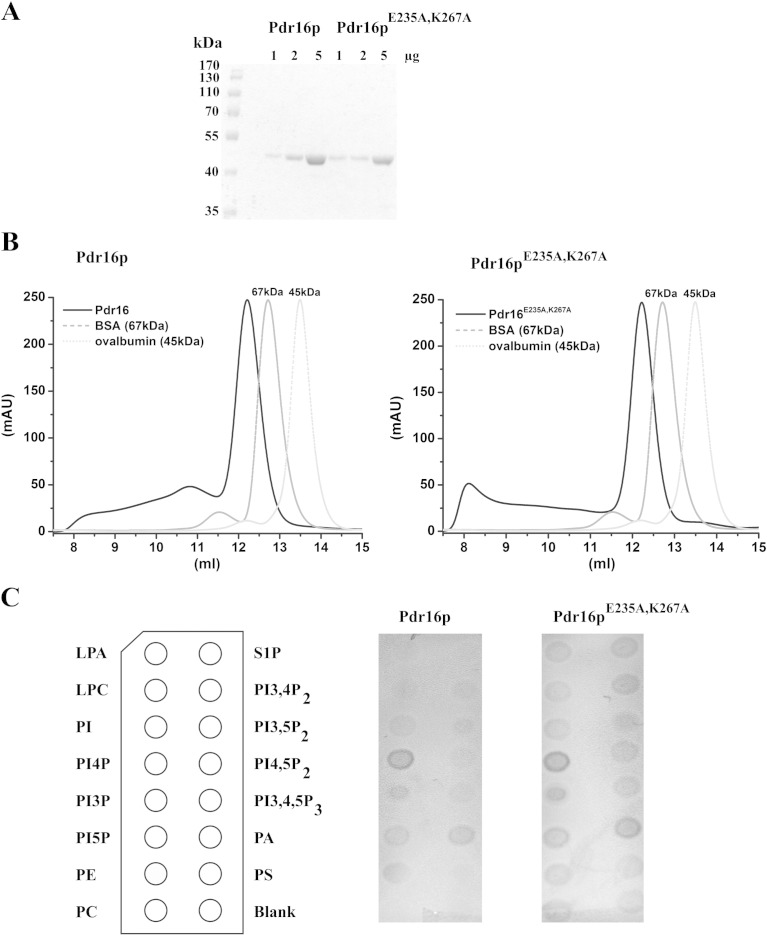
Comparison of Pdr16p and its mutant defective in PI binding, Pdr16p ^E235A, K267A^. A: Pdr16p and Pdr16p ^E235A, K267A^ can be stably expressed in *E. coli* and purified using Ni-NTA beads. SDS-PAGE gel of indicated amounts of purified recombinant Pdr16 and Pdr16 ^E235A, K267A^ proteins was stained with Coomassie blue. B: Both, Pdr16p and Pdr16p ^E235A, K267A^ are present as homodimers in *E. coli* extracts. The purified recombinant Pdr16 proteins were subjected to size exclusion chromatography on a Superose™ 12 10/300 GL column, using bovine serum albumin (BSA) and ovalbumin as protein molecular weight standards. Both Pdr16 proteins elute with an apparent molecular mass of approximately 90 kDa. Predicted molecular mass of Pdr16p monomer is 40.7 kDa. C: Lipid–protein overlays using wild-type Pdr16p and Pdr16p ^E235A, K267A^, respectively, showed the preferred affinity of both proteins to PA and PI4P immobilized on the nitrocellulose membranes. Abbreviations: LPA, Lysophosphatidic acid; LPC, Lysophosphocholine; PI, Phosphatidylinositol; PI4P, Phosphatidylinositol (4) phosphate; PI3P, Phosphatidylinositol (3) phosphate; PI5P, Phosphatidylinositol (5) phosphate; PE, Phosphatidylethanolamine; PC, Phosphatidylcholine; S1P, Sphingosine 1-Phosphate; PI3,4P_2_, Phosphatidylinositol (3,4) bisphosphate; PI3,5P_2_, Phosphatidylinositol (3,5) bisphosphate; PI4,5P_2_, Phosphatidylinositol (4,5) bisphosphate; PI3,4,5P_3_, Phosphatidylinositol (3,4,5) trisphosphate; PA, Phosphatidic acid; PS, Phosphatidylserine; Blank, no lipid spotted.

The following experiments indicate that the mutant Pdr16p^E235A, K267A^ maintains the overall structure similar to wild-type Pdr16p: (i) both proteins can be stably expressed and purified from bacteria (Fig. 3A) and yeast; (ii) like wild-type Pdr16p, recombinant mutant Pdr16p^E235A, K267A^ purifies from *E. coli* as a dimer (Fig. 3B); (iii) both wild-type and mutant Pdr16p^E235A, K267A^ preferentially associate with the same phospholipids, PI4P and PA, in protein-phospholipid overlay assays (Fig. 3C). We would like to point out that the lipid overlay assay monitors a different property of the protein compared to the lipid binding assay using HL60 cells. When using permeabilized HL60 cells the lipid is extracted from the membrane and inserted into the lipid binding pocket of Pdr16p. In the lipid overlay assay we assessed the ability of the external protein surface to interact with the lipids. Mutations (E235A and K267A) were made that affect the ligand binding pocket. Taken together, our results show that in the mutant protein only the lipid binding in the hydrophobic cavity is disrupted (Fig. 2) but not the surface properties of the protein (Fig. 3C).

### 3.3 Pdr16p^E235A, K267A^ mutant defective in PI binding is not able to provide protection against azole antimycotics

**Fig. 4:**
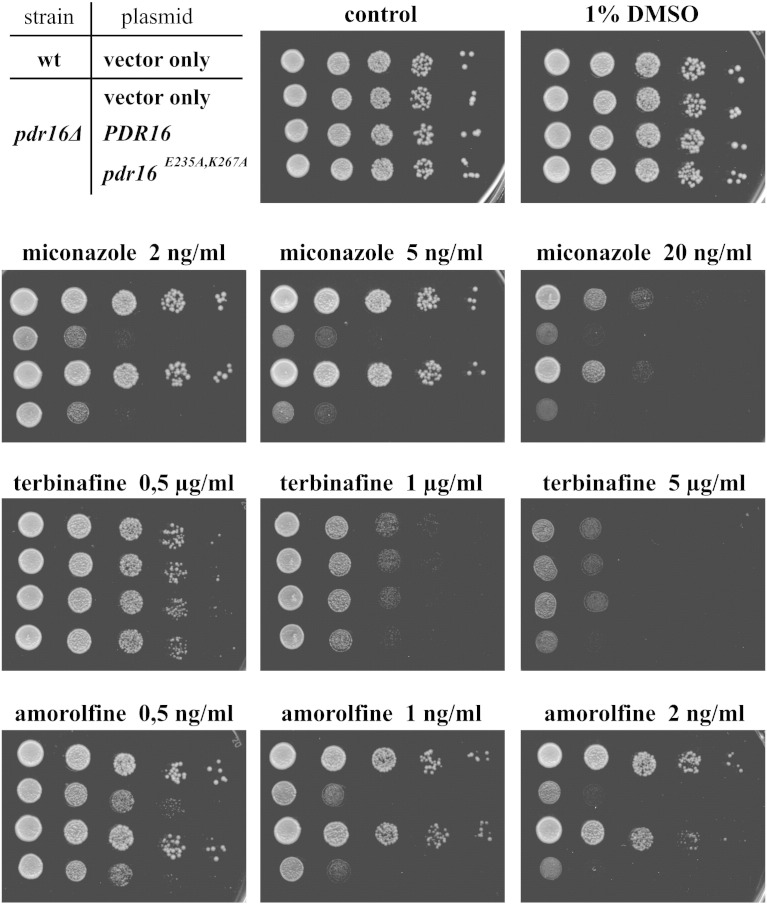
Susceptibility of yeast strains overexpressing wild-type Pdr16p and PI binding deficient Pdr16p ^E235A, K267A^ to ergosterol biosynthesis inhibitors. Wild-type (WT) FY1679-28c strain and *pdr16Δ* strain transformed with empty cloning vector YEplac181 (vector only) and vector overexpressing *PDR16* or *pdr16*^E235A, K267A^ were pre-grown on YNB-LEU media overnight, diluted serially 10 times and plated on YEPD media containing indicated concentrations of sterol biosynthesis inhibitors miconazole, terbinafine, and amorolfine. Plates were incubated at 28 °C and assayed after 2 days.

To test whether mutant Pdr16p^E235A, K267A^ is able to provide protection against azole antimycotics similar to wild-type Pdr16p we inserted *PDR16* and mutated *pdr16* ^*E235A*,^ *^K267A^* allele into an episomal multicopy plasmid. Expression of *PDR16* and its mutant allele, defective in PI binding were regulated by native *PDR16* promoter. These plasmids, together with the empty cloning vector were transformed into yeast strain containing deletion of the chromosomal copy of the *PDR16* gene. The resulting yeast strains were challenged with the presence of azole antimycotic, miconazole (Fig. 4). The experiment confirmed the increased susceptibility of the *pdr16Δ* strain towards miconazole compared to wild-type cells [11], [12]. Re-introduction of the wild-type *PDR16* allele on a plasmid provides protection to the *pdr16Δ* strain towards miconazole that is similar to the level of protection provided by the chromosomal copy of *PDR16*. However, overexpression of the *pdr16* ^*E235A*,^ *^K267A^* mutant allele provides no protection against miconazole to the *pdr16Δ* cells. Minimal inhibitory concentration (MIC) for wild-type and the *pdr16Δ* strain containing *PDR16* on a multicopy plasmid was 20 ng/ml of miconazole, whereas MIC for the *pdr16Δ* strains containing the empty cloning vector or the mutated *pdr16* ^*E235A*,^ *^K267A^* allele was 2 ng/ml of miconazole. In addition to miconazole we assessed susceptibility of the above mentioned yeast strains to terbinafine and amorolfine, two drugs that inhibit yeast ergosterol biosynthetic pathway at different steps than azoles. Terbinafine is an allylamine derivative that specifically inhibits fungal squalene epoxidases (Erg1p in *S. cerevisiae*) converting squalene to 2,3-oxidosqualene [30], [31]. Morpholine fungicide amorolfine affects two targets in the ergosterol pathway: delta 14 reductase (Erg24p) and delta 8–delta 7 isomerase (Erg2p) [32], [33]. Our results show that *pdr16Δ* strain and also *pdr16Δ* strain containing *pdr16* ^*E235A*,^ *^K267A^* allele defective in PI binding are more susceptible to amorolfine but not terbinafine compared to their parental wild-type strain FY 1679-28c. These results indicate that yeast cells without the functional Pdr16p are more susceptible to drugs that specifically affect relatively later steps of the ergosterol biosynthetic pathway.

**Fig. 5:**
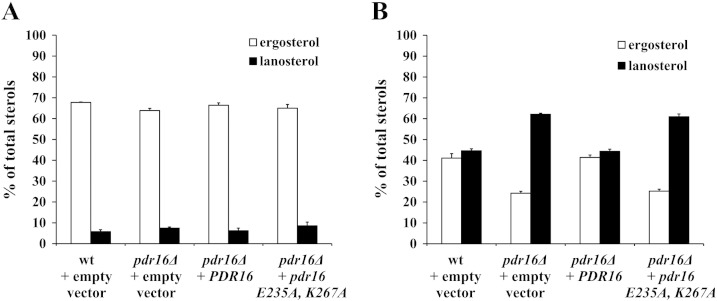
Sterol composition of the *pdr16Δ* strain overexpressing wild-type Pdr16p and PI binding deficient Pdr16p ^E235A, K267A^. *pdr16Δ* strain containing either the empty multicopy cloning vector YEplac181, the wild-type *PDR16* allele in YEplac181 plasmid or mutant *pdr16*^E235A, K267A^ allele in YEplac181 plasmid together with the parental wild-type strain FY1679-28c were pre-grown in synthetic yeast media without leucine as required for plasmid selection. Subsequently, they were grown for 6 hours at 28 °C in YEPD media without miconazole (A) or with sub-inhibitory concentration of miconazole (5 ng/ml) (B). Following extraction non-saponifiable lipids were analyzed by HPLC as described in Materials and methods. Total sterols represent ergosterol and its precursors including lanosterol, and squalene. Data represent mean ± S.E.M. from three experiments.

Next, we measured the relative amounts of ergosterol and lanosterol in the following strains challenged by sub-inhibitory concentration of miconazole: (a) wild-type; (b) *pdr16Δ*; (c) *pdr16Δ* expressing wild-type *PDR16* gene from a multicopy plasmid; (d) *pdr16Δ* expressing mutant *pdr16* ^*E235A*,^ *^K267A^* allele from a multicopy plasmid. The results show that in the absence of miconazole the relative amounts of ergosterol and lanosterol were the same in all four strains (Fig. 5A). In the presence of sub-inhibitory concentrations of miconazole, the *pdr16Δ* cells displayed increased accumulation of lanosterol at the expense of the final product of the sterol biosynthetic pathway, ergosterol (Fig. 5B). Accumulation of lanosterol, an early sterol precursor in the ergosterol biosynthetic pathway that is substrate for lanosterol 14α-demethylase, the major target of azole antifungals [34], [35] serves as an indicator of the functionality of ergosterol biosynthetic pathway. Introduction of the *PDR16* gene on a multicopy plasmid into the *pdr16Δ* cells resulted in reversion of the sterol profile of the *pdr16Δ* strain to that of a parental wild-type. On the other side, introduction of the PI binding defective Pdr16p ^E235A, K267A^ resulted in no change in neutral lipids profile compared to *pdr16Δ* cells (Fig. 5B).

## 4 Discussion

*S. cerevisiae* Sec14 homologues form a diverse group of proteins with distinct subcellular localizations [17] and diverse functions related to lipid metabolism, membrane trafficking and phosphoinositide mediated signaling (reviewed in [8], [36]). The unified feature of these proteins is their ability to transfer PI between membranes in *in vitro* systems*.* Therefore, they are classified as PI transfer proteins [9]. One of these proteins is Pdr16p (also known as Sfh3p). Pdr16p is required for resistance of yeast cells to all azole antifungals tested [9], [10], [11]. To understand the role of Pdr16p in providing protection to azole antifungals we generated mutant protein defective in PI binding. To demonstrate that this mutant Pdr16p ^E235A, K267A^ is unable to bind PI we employed *in vitro* lipid binding assay in which purified lipid transfer protein is co-incubated with radiolabeled permeabilized HL60 cells. The results (Fig. 2) show that the mutant Pdr16p ^E235A, K267A^ binds very little PI compared to the wild-type Pdr16p. Otherwise, Pdr16p ^E235A, K267A^ behaves similarly to wild-type Pdr16p in conformation studies (Fig. 3B) and protein-lipid overlay (Fig. 3C) studies. Thus, it can be considered that Pdr16p^E235A, K267A^ still retains the overall structural features of wild-type Pdr16p. This PI binding defective mutant Pdr16p ^E235A, K267A^ was unable to provide protection against azole antifungal, miconazole (Fig. 4). Upon miconazole treatment increased amounts of lanosterol, the substrate for miconazole target enzyme, lanosterol-14α-demethylase, can be seen in *pdr16Δ* cells compared to wild-type cells. Expression of the *PDR16* gene in *pdr16Δ* cells reversed lanosterol/ergosterol ratio to that of wild-type cells. However, overexpression of PI binding deficient mutant *PDR16* ^*E235A*,^ *^K267A^* did not lower the high levels of lanosterol in *pdr16Δ* cells (Fig. 5). Thus, we conclude that *in vitro* observed PI binding by Pdr16p [9], [29] is essential for its *in vivo* function in modulating sterol homeostasis in yeast.

The HL60 binding assay demonstrated an unexpected characteristic of Pdr16p and Pdr16p ^E235A, K267A^ mutant, the ability to bind cholesterol. It is the first example, to our knowledge, when a phosphatidylinositol binding protein strongly associates with sterol molecules. It remains to be established, however, whether sterol can be taken into the lipid cavity of Pdr16p. The difference between the lipid binding pocket of Pdr16p and other members of the Sec14 group of yeast PITPs for which the structure is known, Sec14p and Sfh1p, supports such a possibility. Upon solving the structure of Pdr16p, Yuan et al. [19] entertained the hypothesis that the much larger lipid pocket of Pdr16p compared to Sec14p or Sfh1p may accommodate some new substrates in addition to PI or PC. Recently, Maeda et al. [37] tested all members of the Sec14 family of yeast PITPs as well as all yeast Osh (oxysterol-binding homology) proteins for their ability to bind lipids *in vivo* using an integrated approach combining protein fractionation and lipidomics. Unfortunately, they were not able to produce any data on Pdr16p lipid binding due to an inefficient recovery of TAP tagged Pdr16p from yeast cell lysates.

While preparing this manuscript, PI binding property of Pdr16p (Sfh3p) was identified as an essential feature for function of Pdr16p as a modulator of lipid droplet neutral lipid utilization [29]. Authors proposed that the modulation of neutral lipid utilization from lipid droplets by Pdr16p is also behind the observed azole susceptibility phenotypes associated with *pdr16Δ* mutants [11], [12]. We do not see such a direct correlation between observed modulation of neutral lipids utilization [29] and enhanced azole susceptibility of *pdr16Δ* cells for the following reasons: (i) there is also decreased biosynthesis of ergosterol in *pdr16Δ* cells compared to wild-type cells when challenged with azoles [11]; (ii) over-expression of the *PDR17* gene can complement the enhanced azole susceptibility of *pdr16Δ* cells [11] contrary to the observed fact that no yeast Sec14-like PITP, including Pdr17p, can fulfill the Pdr16p role in control of lipid droplet utilization [29]. Thus, we think that Pdr16p has a much broader role in ergosterol homeostasis than just modulation of neutral lipid utilization.

Nile et al. [38] successfully explored nitrophenyl(4-(2-methoxyphenyl)piperazin-1yl)methanones (NPPMs) as small molecule inhibitors of the major yeast PITP, Sec14p. Their data indicated that NPPMs load into the Sec14p hydrophobic pocket during the phospholipid exchange cycle. Their work established PITPs as pharmacological targets to modify PIP signaling in eukaryotic cells. Our finding that PI binding deficient Pdr16 mutant is ineffective in providing protection against azole antifungals opens the possibility for chemical intervention to modify Pdr16p mediated azole resistance in yeast.

**Fig. 6:**
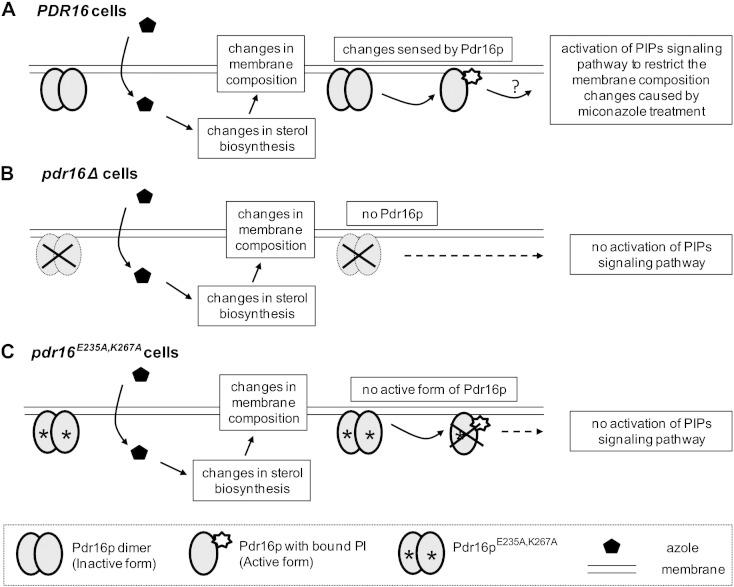
A model for Pdr16p as a sensor of membrane lipid composition. A. Pdr16p senses the changes in membrane lipid composition caused by the presence of miconazole. These changes result in PI binding, monomerization of Pdr16p and relaying the information to the signaling pathway to modify sterol metabolism to protect the cells against azoles. B. Without Pdr16p no signal is generated and the cells are more sensitive to azole antimycotics. C. Pdr16p ^E235A, K267A^ mutant is defective in PI binding and thus unable to relay the information of membrane lipid composition changes to the signaling pathway. As a result the cells are hypersensitive to azoles.

Based on our recent results (this paper and [11]) we propose two mechanisms for the function of Pdr16p in providing protection against azole antifungals. Firstly, Pdr16p could be required for effective functioning of the ergosterol biosynthetic pathway by helping to shuttle sterols or their intermediates *via* intermembrane contact sites or alternatively, between biosynthetic enzymes or complexes. This hypothetical function of Pdr16p is based mostly on the ability of Pdr17p, a known component of intermembrane contact sites for transfer of PS [39], [40], to substitute for Pdr16p in providing protection against azole antifungals [11]. Interestingly, another identified essential component of the well studied intermembrane contact site protein complex required for PS transport from ER to endosomes is Stt4 phosphatidylinositol-4-kinase [41], [42]. Current thoughts on the function of Sec14-like PITPs consider these as PI presentation proteins to PI kinases [43], [44]. Thus, the fact that PI binding deficient mutant does not provide protection against azole antifungals fits nicely into this scenario. Secondly, we could consider Pdr16p to be a hypothetical sensor of membrane sterol composition (Fig. 6). It could sense the changes in membrane lipid composition upon azole treatment and relays this information *via* presentation of PI to a PI kinase to activate a signaling cascade leading to modification of sterol metabolism. Whether any of these scenarios represent the real mechanism how Pdr16p functions remains to be experimentally verified. We believe that at least some aspects of these models can be tested in the near future.

## 5 Conclusions

We generated a Pdr16p^E235A, K267A^ mutant defective in PI binding. Using an *in vitro* lipid binding assay based on presentation of radiolabeled lipids in permeabilized HL60 cells to the lipid transfer proteins we have shown that Pdr16p is able to bind sterols in addition to PI. Mutant Pdr16p^E235A, K267A^ is defective in PI binding; it binds almost exclusively cholesterol instead. PI binding deficient Pdr16p^E235A, K267A^ is not able to fulfill the role of Pdr16p in protection against azole antifungals, providing evidence that PI binding of Pdr16p is critical for its function in modulation of sterol metabolism in response to the presence of azoles.
